# Impact of Hydrophilic Modification of Synthetic Dialysis Membranes on Hemocompatibility and Performance

**DOI:** 10.3390/membranes12100932

**Published:** 2022-09-26

**Authors:** Adam M. Zawada, Thomas Lang, Bertram Ottillinger, Fatih Kircelli, Manuela Stauss-Grabo, James P. Kennedy

**Affiliations:** 1 Product Development, Fresenius Medical Care Deutschland GmbH, 66606 Sankt Wendel, Germany; 2 Global Biomedical Evidence Generation, Fresenius Medical Care Deutschland GmbH, 61352 Bad Homburg, Germany; 3 Ottillinger Life Sciences, 85649 Brunnthal, Germany; 4 Medical Information and Education (EMEA), Fresenius Medical Care Deutschland GmbH, 61352 Bad Homburg, Germany

**Keywords:** dialysis, performance, hemocompatibility, membrane, protein fouling, end-stage kidney disease, polyvinylpyrrolidone, hydrophilicity

## Abstract

The dialyzer is the core element in the hemodialysis treatment of patients with end-stage kidney disease (ESKD). During hemodialysis treatment, the dialyzer replaces the function of the kidney by removing small and middle-molecular weight uremic toxins, while retaining essential proteins. Meanwhile, a dialyzer should have the best possible hemocompatibility profile as the perpetuated contact of blood with artificial surfaces triggers complement activation, coagulation and immune cell activation, and even low-level activation repeated chronically over years may lead to undesired effects. During hemodialysis, the adsorption of plasma proteins to the dialyzer membrane leads to a formation of a secondary membrane, which can compromise both the uremic toxin removal and hemocompatibility of the dialyzer. Hydrophilic modifications of novel dialysis membranes have been shown to reduce protein adsorption, leading to better hemocompatibility profile and performance stability during dialysis treatments. This review article focuses on the importance of performance and hemocompatibility of dialysis membranes for the treatment of dialysis patients and summarizes recent studies on the impact of protein adsorption and hydrophilic modifications of membranes on these two core elements of a dialyzer.

## 1. Introduction

The global prevalence of end-stage kidney disease (ESKD) is rising steadily, mainly caused by the increasing prevalence of ESKD risk factors such as hypertension and diabetes mellitus, higher life expectancy of the general population and better survival of ESKD patients due to improved treatment options [1,2]. While kidney transplantation is the preferred treatment option for eligible ESKD patients, most patients depend on a renal replacement therapy [2]. This therapy can be performed at home with peritoneal dialysis or by extracorporeal treatments, such as low- and high-flux hemodialysis (HD), including low dialysate flow daily HD [3], or hemodiafiltration (HDF), which are the predominant treatment options for patients with ESKD [2,4,5]. In these extracorporeal treatments, a dialyzer replaces the function of the malfunctioning kidney, that is, elimination of a wide range of uremic toxins, e.g., ß2-microglobulin, urea, uric acid, or creatinine, and of excess fluid, while preventing loss of essential proteins, such as albumin [6,7]. This function of a dialyzer is called the performance and is generally described by clearance and sieving coefficient values in the instructions for use of the manufacturers. Performance factors are primarily influenced by the dialyzer membrane, including its composition, membrane morphology and structure (e.g., mean pore size, pore size distribution, surface area, membrane thickness) and adsorptive properties [8,9,10,11,12,13]. Besides strong performance, hemocompatibility is another core element of a dialyzer. Contact of human blood to artificial surfaces of the dialyzer may activate the immune system, leading to complement activation, coagulation and inflammation, with negative clinical consequences for the patients [14,15,16,17]. Additionally, here, the membrane has the strongest effect on the hemocompatibility profile of the dialyzer, as it has the largest contact surface with the patients’ blood during dialysis.

During hemodialysis treatment, the adsorption of plasma proteins to the blood-side surface of the dialyzer membrane can strongly impact both performance as well as the hemocompatibility profile of the dialyzer. In the present review we discuss the two core properties of a dialyzer and show how protein adsorption impacts performance and hemocompatibility. We summarize recent findings how hydrophilic membrane modifications reduce protein adsorption and improve the performance and hemocompatibility profile of a dialyzer. Given that hydrophilic modifications are primarily applied for synthetic dialysis membranes, such as polysulfone (PS) or polyethersulfone (PES) membranes, this review article mainly focuses on this type of membranes and modifications. Due to their innate hydrophilicity, such modifications are not required for membranes containing or made from natural materials such as cellulose and its derivatives. Therefore, such membranes are not in the scope of this present review. For further details about structures and features of other types of synthetic (e.g., polyacrylonitrile (PAN), polymethylmethacrylate (PMMA), polyester polymer alloy (PEPA), ethylene-vinyl alcohol co-polymer (EVAL)), cellulose-based or composite membranes, the interested reader is referred to excellent reviews in this field [9,10,11,12,13,18,19,20].

## 2. The Importance of Dialyzer Performance and Hemocompatibility for the Treatment of ESKD Patients

Patients with ESKD undergoing hemodialysis treatment are a complicated patient population with many comorbidities, as investigated in various international cohort studies [1,5,21,22,23]. Cardiovascular complications are a major problem among this patient population, and a leading cause of death [21,24,25,26,27]. The mortality rate in this population is approx. 6 times higher than in the general population [28].

A pivotal reason for the high rate of complications and reduced life expectancy of patients with ESKD is the accumulation of uremic toxins in the blood. Performance characteristics, i.e., removal of those uremic toxins which accumulated due to the loss of kidney function, is a critical feature of a dialyzer that has impacts on the ESKD-related complications [29]. Although the dialyzer consists of many different parts, such as housing, flange caps or blood/dialysate ports, the membrane is the main determinant for the performance characteristics of the dialyzer. Special focus has been placed on the removal of middle molecules, represented by markers such as ß2-microglobulin (~12 kDa), given that middle molecule accumulation has been associated with increased inflammation, cardiovascular risk and mortality among dialysis patients [29,30,31,32,33]. During the evolution of membranes, pore size increased to allow elimination of such larger uremic toxins. However, such increase in the pore size of the membranes also increases the risk to lose essential larger size proteins such as for example albumin (~68 kDa). Repeated protein loss may lead to the development of malnutrition, which is associated with increased mortality among ESKD patients [34,35,36]. Therefore, albumin has become an established key parameter of nutritional status of hemodialysis patients and has also been used in studies investigating protein leakage into the dialysate [37]. Thus, the performance of a dialyzer is not sufficiently described by its clearance or removal of middle size toxins and should consider its sieving properties overall, including its permeability cut-off for larger size proteins.

A further important aspect in achieving favorable patient outcomes is the dialyzer’s hemocompatibility. Contact of blood with non-body surfaces triggers a wide range of reactions, including immune cell activation, coagulation and activation of the complement system. The complement system is part of human innate immunity, modulating the immune response and promoting the clearance of invading pathogens as well as the removal of damaged or dying cells, immune complexes, and cell debris [38,39,40]. To this end, the complement system releases complement factors and induces cytokines as well as coagulation factors [15,41]. In parallel with complement activation, PMN elastase as a marker of inflammation is released from leucocytes in patients on hemodialysis [42,43,44]. Of note, other markers of inflammation, oxidative stress, or of tissue damage such as hCRP, interleukin-6, tumor necrosis factor-α, myeloperoxidase, or troponin-T showed a positive correlation with the accumulation of uremic toxins, such as ß2-microglobulin, pointing towards the importance of strong dialyzer performance in the context of hemocompatibility [33]. Moreover, these inflammatory markers were associated with makers of cardiovascular disease such as carotid intima-media thickness and the ankle-arm blood pressure index [33,45,46,47,48,49]. Such associations were also seen with regard to complement activation; in a prospective randomized study with 260 hemodialysis patients [50], higher baseline complement factor C3 levels were associated with subsequent cardiovascular events over the following 12 months (cox-regression analysis: hazard ratio 1.20 [1.01–1.42] per 0.1 mg/mL). Furthermore, an observational clinical study with 55 patients on hemodialysis found that those patients who developed cardiovascular events during a maximum follow-up of 45 months (*n* = 17) had significantly increased C3d/C3 levels 30 min after hemodialysis start (*p* < 0.05), as compared to those patients who did not develop cardiovascular events during follow-up (*n* = 38) [51]. Additionally, those patients who experienced a cardiovascular event during follow-up had a higher pro-inflammatory and pro-thrombotic response during dialysis treatment than those patients who did not experience a cardiovascular event (cardiovascular event group: IL-6/IL-10-ratio significantly higher at 60 min [*p* < 0.05], TNF-α levels significantly higher at 180 min [*p* < 0.05], von Willebrand factor significantly higher at 180 and 240 min after dialysis start [*p* < 0.05]). An ex vivo dialysis model found that this C3d/C3 complement activation contributed to the pro-inflammatory and pro-thrombotic response (induction of TNF-α levels, IL-6/IL-10-ratio and von Willebrand factor levels), pointing towards causal role of complement activation in inflammation and cardiovascular disease [51]. Further indication for a causal role of the complement system in inducing cardiovascular events is coming from animal studies, where the inhibition of complement C5a activation (via an complement C5a receptor antagonist) significantly reduced arterial plaque formation (lesion size and lipid content) in atherosclerosis prone mice by ~40% (*p* < 0.05) [52]. In addition to the activation of the complement system, contact of blood with non-body surfaces activates platelets and the coagulation system. Typically, platelet counts show a drop during the dialysis session while thrombin-antithrombin-complex (TAT) concentrations increase despite systemic anticoagulation with heparin, as seen in a recent clinical study [42]. As the activation of the coagulation system during dialysis may contribute to the high cardiovascular event rate of patients with ESKD, dialyzers hemocompatibility need also to address the coagulation activation path. Comparable to dialyzer performance, dialyzers’ hemocompatibility profile is mainly determined by the membrane, given that it has the largest surface which comes in contact with patients’ blood. Different types of membranes exist, which have a different hemocompatibility profile. Most current dialyzers use synthetic membranes with polysulfone (PS) or polyethersulfone (PES) polymers. These polymers are associated with less transient complement activation and drops in leukocyte counts than cellulose-based membranes, so that their hemocompatibility is superior to the latter [19,53,54,55,56,57]. Besides the membrane material, other factors such as geometry, the amount and arrangement of hydrophilic and hydrophobic areas on the membrane surface or the membrane surface charge (zeta potential) contribute to the hemocompatibility profile of the membrane [54].

## 3. Impact of Membrane Fouling on Dialyzers’ Performance and Hemocompatibility

During a hemodialysis treatment, the contact of blood components with the membrane of the dialyzer leads to the formation of a secondary membrane, which is composed of plasma proteins adsorbed to the dialyzers’ membrane. This secondary membrane is composed of a milieu of different plasma proteins, such as albumin or fibrinogen, and has strong implications for the performance and hemocompatibility profile of a dialyzer.

**Figure 1:**
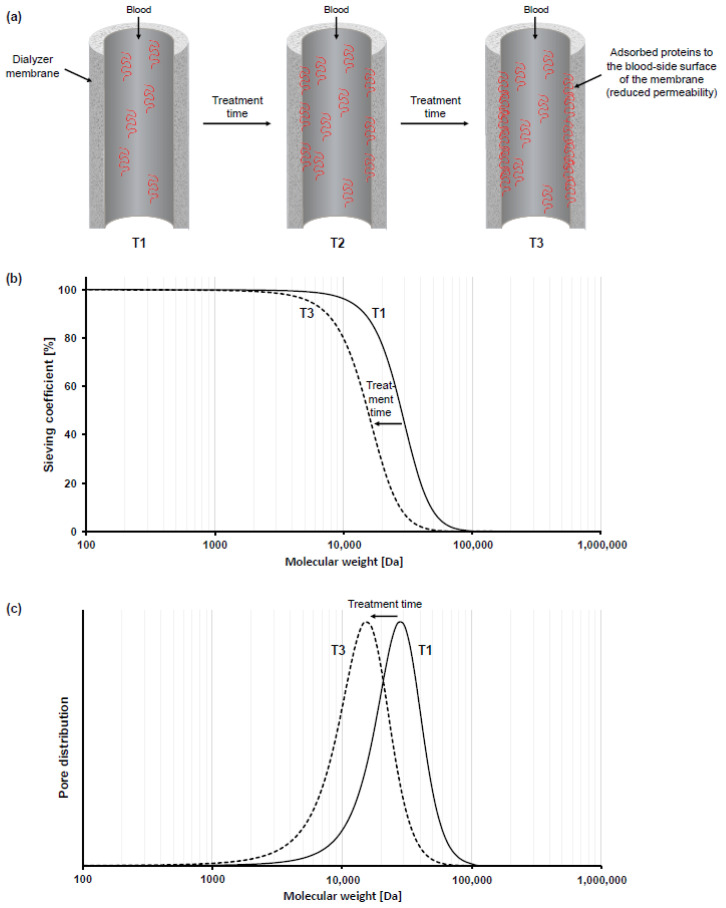
Schematic illustration of the impact of protein adsorption on molecular weight retention curves and effective pore size distribution. Protein adsorption to the membrane during dialysis treatment leads to a reduction in permeability of the membrane (**a**). The secondary membrane leads to a shift in molecular weight retention curves (**b**) and the effective pore size distribution (**c**).

To describe the performance characteristics of a dialyzer, manufacturers present clearance values for different solutes such as for urea, creatinine, or phosphate in the respective instructions for use. However, it is important to emphasize that those clearance values are measured in an aqueous solution, which does not contain any plasma proteins. In contrast, during hemodialysis treatment, plasma proteins in the patients’ blood lead to a buildup of a secondary membrane within minutes, which adds an additional barrier to uremic solute exchange [58,59,60,61,62,63,64]. This accumulation of adsorbed proteins at the membrane surface significantly reduces dialyzer performance as compared to the pristine membrane or “native performance” (Figure 1a).

That toxin removal capacity of a dialyzer is not a constant value but diminishes during dialysis treatment due to the adsorption of plasma proteins and the buildup of a secondary membrane is known for many years. More than three decades ago, Röckel et al., showed that during the first 10 min of hemofiltration, the investigated polysulfone dialyzer was permeable to substances up to 66 kDa, which reduced to less than 30 kDa within 20 min [59]. Here, the impact of the secondary membrane is more pronounced on the permeability for larger molecules, such as proteins, than on smaller solutes such as urea or Vitamin B_12_ [59,60,61,62]. In a recent experimental study with three different synthetic dialyzers, we confirmed these previous findings, by measuring sieving coefficient changes over 240 min of the three proteins albumin, myoglobin and β2-microglobulin for three different synthetic dialyzers [58]. For all dialyzers, strongest decrease in sieving coefficients was found in the first 20–30 min of plasma recirculation, with a stronger decrease for larger molecules than for smaller proteins (e.g., 94% mean relative decrease in albumin [~68 kDa] sieving coefficient vs. 57% mean relative decrease in myoglobin [~17 kDa] sieving coefficient over 240 min and 8% mean relative decrease in β2-microglobulin [~12 kDa] sieving coefficient over 120 min; *n* = 3 for each of the three dialyzers). When analyzing molecular weight retention curves, such curves show a typical shift towards lower molecular weight during the treatment [58,60,63]. Moreover, the effective pore size of the membrane also shows a typical reduction after contact of plasma proteins to the membrane. An illustration of this phenomenon is schematically presented in Figure 1b (molecular weight retention curves) and Figure 1c (effective pore size). Of note, the characteristics (e.g., thickness, density, composition) of the protein layer formed on the membrane surface impacts the extent of performance reduction and shift in molecular weight retention curves [58,60]. Membranes with a thicker protein layer have a stronger reduction in performance, which is associated with stronger reduction in the effective pore size of the dialyzer membranes [58,60]. Data from a recent randomized controlled trial with 52 hemodialysis patients treated with three different synthetic dialyzers in a cross-over design, support these findings, showing superiority in β2-microglobulin removal rate for that dialyzer that had the lowest secondary membrane formation (75.5% vs. 74.0% and 72.7%; *p* = 0.0216 and *p* < 0.0001, respectively) [37]. Therefore, these blood-membrane interactions influence the efficacy of the dialysis treatment and have to be taken into considerations beyond the clearance values presented in the instructions for use of the dialyzers.

Besides the impact on performance, protein adsorption to the membrane also strongly impacts the hemocompatibility profile of a dialyzer. The adsorption of proteins to the artificial surface triggers conformational changes or denaturation of protein structures which lead to their activation [16]. Contact activated proteins can then trigger different pathways, such as activation of immune cells as well as of the complement and coagulation system [16,65,66]. In addition to the amount of bound proteins and the degree of contact activation, the type of bound proteins to the membrane may impact the immune response. For example, it is well known that the adsorption of fibrinogen to the membrane triggers binding and activation of platelets [16,67,68]. Therefore, to characterize the hemocompatibility profile of a dialyzer, quantitative and qualitative evaluation of the adsorbed proteins to the membrane surface may help to understand differences in hemocompatibility of different dialyzers. Recently, we characterized secondary membrane formation and hemocompatibility profile of different synthetic and cellulose-based dialyzers which are commonly used for the treatment of dialysis patients [54,58,69]. In an in vitro approach, secondary membrane formation was characterized by measuring changes in albumin sieving coefficients over 240 min, as a surrogate of protein adsorption. Moreover, platelet loss and the activation of the complement system was measured by determining levels of the complement factors C3a, C5a and sC5b-9 in an in vitro system with human whole blood. In line with previous literature, those dialyzers which showed a stronger secondary membrane formation induced higher platelet loss (dialyzer with lowest secondary membrane formation: −225% less platelet loss than a reference dialyzer; dialyzer with strongest secondary membrane formation: +95% more platelet loss than a reference dialyzer; *p* < 0.05) and complement activation (dialyzer with lowest secondary membrane formation: −39% [C3a], −57% [C5a] and −59% [sC5b-9] lower complement activation than a reference dialyzer; dialyzer with strongest secondary membrane formation: +56% [C3a], +268% [C5a] and +207% [sC5b-9] higher complement activation than a reference dialyzer; *p* < 0.001, *p* < 0.01 and *p* < 0.01, respectively) than dialyzers with lower secondary membrane formation [54,58,69]. Data from a recent randomized controlled trial with 70 hemodialysis patients support these experimental findings in terms of complement activation (significantly lower sC5b-9 complement activation 60 min after dialysis start with synthetic dialyzer with lower secondary membrane formation vs. synthetic dialyzer with higher secondary membrane formation; *p* = 0.021), indicating that protein adsorption to the membrane during hemodialysis treatment is a key determinant for the hemocompatibility profile of the dialyzer [42].

## 4. Reduction in Membrane Fouling by Hydrophilic Modifications

**Figure 2:**
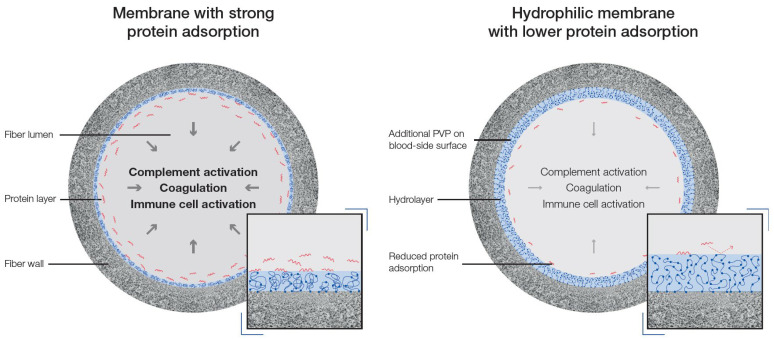
Schematic illustration of complement activation, coagulation and immune cell activation by a membrane with strong protein adsorption as compared to a hydrophilic membrane with lower protein adsorption. Increase in hydrophilicity can be achieved by an increased content of the hydrophilic agent polyvinylpyrrolidone (PVP) on the blood-side surface of the membrane, which reduces protein adsorption via repulsive hydration force of the formed water layer. Protein binding to the membrane leads to conformational changes or denaturation of protein structures which can subsequently trigger complement activation, coagulation, and immune cell activation.

Over the course of the last several decades, dialyzer membrane research has focused on improving both performance and hemocompatibility. As protein adsorption to the membrane impacts both–performance and hemocompatibility–membrane modifications with the aim to reduce secondary membrane formation during dialysis treatment were in the focus of latest dialyzer development. For synthetic membranes, such as polysulfone- or polyethersulfone-based membranes, polyvinylpyrrolidone (PVP) is commonly used as a hydrophilic agent. PVP has good physiological inertness and reduces protein adsorption via repulsive hydration force of the formed water layer [67,70,71,72,73,74]. Wang et al. fabricated polyethersulfone-based membranes with increased PVP content and found that those membranes with higher PVP content showed stronger water adsorption (membrane with 0% PVP content: ~90 μg/cm^2^ water adsorption; membrane with 6% PVP content: ~160 μg/cm^2^ water adsorption) and were associated with reduced albumin adsorption (membrane with 0% PVP content: ~120 μg/cm^2^ albumin adsorption; membrane with 6% PVP content: ~80 μg/cm^2^ albumin adsorption) as well as increased blood coagulation time (membrane with 0% PVP content: ~40 s activated partial thrombin time; membrane with 6% PVP content: ~95 s activated partial thrombin time) [74]. In line, Zhu and colleagues prepared and characterized polysulfone membranes with different amounts of PVP and found that membranes with higher PVP content showed lower protein adsorption, reduced platelet adhesion and deformation as well as improved blood clotting characteristics [70]. Differences in PVP content in polysulfone-based membranes also affect the roughness of the membrane in dry or wet condition and are strong determinants for the swelling of the membrane after contact with water [67,73]. These findings are schematically summarized in Figure 2.

**Figure 3:**
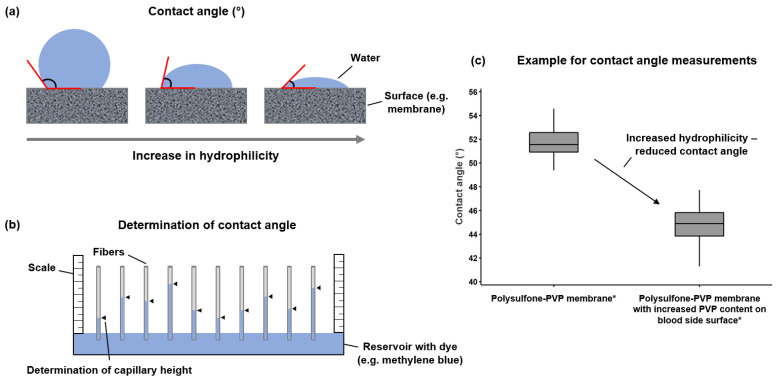
Determination of membrane hydrophilicity with contact angle measurements. Membranes with higher hydrophilicity have a lower contact angle than membranes with a lower hydrophilicity (**a**). To determine contact angle, fibers are placed in a water reservoir containing a dye (e.g., methylene blue) and the capillary height is measured with a scale after a defined time (**b**). Based on the capillary height and other parameters such as membrane geometry, the contact angle can be determined. When comparing two membranes with the same geometry but different hydrophilicity, the more hydrophilic membrane will have a higher capillary height and lower contact angle, as shown exemplarily for two membranes with the same geometry but different hydrophilicity (*n* = 30 membranes, each; *p* < 0.001 (*t*-test)) (**c**). * Shown are a polysulfone-PVP membrane (Helixone^®^*plus* of the FX CorDiax 600 dialyzer, Fresenius Medical Care) and a polysulfone-PVP membrane with increased PVP content on the blood side surface (Helixone^®^*hydro* of the FX CorAL 600 dialyzer, Fresenius Medical Care).

Hydrophilicity of the dialyzer membrane is generally characterized by contact angle measurements. Figure 3 schematically shows the principle and the measurement of contact angle in dialysis membranes. Increasing hydrophilicity of the membrane is associated with a lower contact angle as shown in Figure 3a. To determine contact angle, one end of a hollow fiber is placed for a defined time in a water reservoir containing a dye, for example methylene blue (Figure 3b). Based on the capillary height, measured with a scale, the contact angle can be determined. Here, two membranes with the same geometry but different hydrophilicity will have different capillary heights and different contact angles, as exemplarily shown for two membranes (Figure 3c), a polysulfone-PVP membrane (Helixone^®^*plus* membrane of the FX CorDiax 600 dialyzer (Fresenius Medical Care, Bad Homburg, Germany)) and a polysulfone-PVP membrane with the same geometry but with increased PVP content on the blood-side surface of the membrane (Helixone^®^*hydro* membrane of the FX CorAL 600 dialyzer (Fresenius Medical Care)), which we recently characterized in experimental and clinical studies [37,42,54,58,69]. These studies found that secondary membrane formation of the membrane with increased PVP content on the blood-side surface (as characterized with X-ray photoelectron spectroscopy) was smaller as compared to dialyzers with lower PVP content (membrane with highest PVP content: −0.015 albumin sieving coefficient slope as marker for secondary membrane formation; membrane with lowest PVP content: −0.104 albumin sieving coefficient slope; *p* < 0.001) [54,58,69]. This lower protein adsorption was moreover associated with lower complement activation, lower platelet loss and also lower loss in performance in experimental studies [54,58,69]. These experimental findings were supported by two randomized controlled trials with in total 122 dialysis patients, which found that the reduced secondary membrane formation of the more hydrophilic membrane was associated with efficient removal of small and middle molecules and with a favorable hemocompatibility profile [37,42].

## 5. Maintaining Hydrophilic Modification of Dialysis Membranes

While the increase in PVP content on the blood-side surface of the membrane leads to increased hydrophilicity and subsequently to lower protein fouling and better hemocompatibility as well as performance stability, the PVP must remain on the blood-side membrane surface in order to have an effect. Unfortunately, it has been well established that PVP can be eluted from the membrane during dialysis treatment [75,76]. This section discusses both the potentially undesirable effects of eluted PVP, as well as the predominant factors that lead to the phenomenon.

### 5.1. Undesirable Effects of Elutable PVP

The reduction in PVP content caused by PVP elution comes not only with negative implications for the hemocompatibility profile and performance of the membrane, but PVP may itself have direct negative impacts on the patient. 

It has long been understood that PVP can be taken up by, e.g., macrophages and lead to storage disease by accumulation of PVP in different tissues or organs such as liver, kidneys or lymph nodes. This disorder has been seen in patients who received PVP injections as plasma substitute in former times [77,78], but up to now no data is available which shows that the elution of PVP from dialyzers may lead to a significant accumulation in the patients’ body.

More recently some reports speculated that eluted PVP could be a cause for adverse reactions, such as hypersensitivity reactions or thrombocytopenia, which rarely occur during treatment with synthetic membranes [12,79,80,81,82,83]. Konishi et al. [84] investigated this potential impact of PVP elution on patient reactions by recruiting patients who previously experienced adverse reactions during treatment with synthetic membranes (defined as hypotension, malaise or symptoms of anaphylactic shock). By performing a skin prick test with PVP, the authors found that none of the 7 patients reacted positive on this test. Therefore, the authors concluded that not PVP, but other factors should be causative for these infrequently occurring adverse patient reactions during treatment with synthetic membranes. Despite the suspicion surrounding PVP, there is currently no clinical study which showed a causal relationship between PVP elution and adverse patient reactions. Nevertheless, there is good reason to avoid elution of PVP from the membrane even if it is only to avoid the negative implications for the performance and hemocompatibility profile of the dialyzer. 

### 5.2. Factors Influencing PVP Elution

The polymer backbone of PVP can undergo free-radical oxidation. Blood-side oriented chains of PVP that are especially important for binding water and generating the protein-repulsive layer of PVP-bound water (hydrolayer) are susceptible to polymer chain breaks that leave these chains no longer anchored to the membrane. These unanchored PVP fragments can be eluted from the membrane during dialysis treatment, leaving gaps in the protective hydrolayer of the membrane. Generation of elutable PVP fragments can occur either relatively quickly during high-energy sterilization processes, or more slowly over long periods of time. Additionally, shear stress within the capillary membrane has been shown to influence PVP elution. These factors are considered in more detail below. 

The type of dialyzer sterilization is a strong determinant for PVP elution. For example, gamma sterilization has been discussed to stabilize PVP in the membrane, by crosslinking PVP with the membrane and was shown to induce lower PVP elution than autoclave sterilization [69,76,85]. We recently also investigated PVP elution across six synthetic dialyzers sterilized with gamma, autoclave steam or INLINE steam [69]. In agreement with previous reports, we observed that autoclave steam sterilization was associated with approx. 3.5-fold higher PVP elution than gamma sterilization. Moreover, lowest PVP elution was found for the INLINE steam sterilized dialyzers (*p* < 0.001 vs. gamma and autoclave steam sterilized dialyzers), where all measurements were below the quantification limit of the method. The low PVP elution from membranes that were sterilized with INLINE steam may be explained by the fact that during the sterilization process the membranes are continuously rinsed with steam and sterile water, that allow efficient removal of any PVP generated during the manufacturing process [86]. 

**Figure 4:**
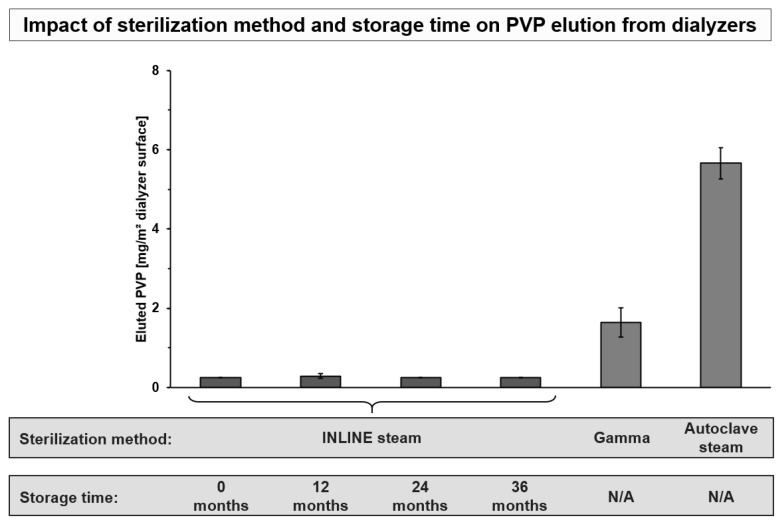
Impact of sterilization method and storage time on PVP elution from dialyzers. Comparison of the amount of eluted PVP in a recirculation system with water for 4 h, as described before [69]. Displayed is the blood-side PVP elution from the INLINE steam sterilized dialyzer FX CorAL 600 (Fresenius Medical Care; *n* = 9) over shelf life as compared to gamma (xevonta Hi 15, B. Braun and ELISIO 17H, Nipro; *n* = 3 each) and autoclave steam (Polyflux 170H, Baxter and Theranova 400, Baxter; *n* = 3 each) sterilized dialyzers, reanalyzed from recently published data [69]. The PVP detection limit for the respective method is 0.5 mg/L; in case of results below detection limit, data are presented as half of the detection limit, as described before [69]. N/A: For these gamma and autoclave steam sterilized dialyzers, no data over shelf life was available; measurement was performed at one time point within their specified shelf life.

Storage time over the shelf life of dialyzers is another determinant for PVP elution from the membranes. Miyata et al. [85] investigated the impact of storage time on PVP elution from autoclave steam and gamma sterilized dialyzers. The authors found a strong correlation between the amount of PVP eluted by washing and the storage period for both dialyzers (r = 0.958, *p* < 0.001 and r = 0.952, *p* < 0.001). Here, oxidation of PVP over time is a factor which leads to the increased PVP elution during storage [85,87]. Therefore, novel membranes have been developed which shall prevent this oxidation and stabilize PVP in the membrane [37,42,54,58,69]. This stabilization was achieved by adding small amounts of the anti-oxidant α-tocopherol to the membrane. In contrast to bioactive membranes, which also use α-tocopherol to achieve therapeutic effects [88,89], the concentration in these novel membranes is much lower, as it just has the aim to stabilize PVP in the membrane. In combination with INLINE steam sterilization, such membranes show no detectable PVP elution [69]. This is also the case when investigating the complete shelf-life of three years of the dialyzers. Figure 4 summarizes these findings on PVP elution and the effects of storage time and different sterilization methods.

Finally, elution of PVP can also be exacerbated through shear stress and filtration, which was investigated by Matsuda et al. [75] in an experimental approach by using a dextran solution as blood substitute. In shear-stress loading experiments up to 144 h, the authors found a correlation between lower PVP retention in the membrane with higher shear-stress loading time and higher magnitude of shear stress. Such results were confirmed by Namekawa et al. [76] showing that increasing shear stress directly increases the elution of PVP. Moreover, the authors investigated the hardness and adsorption force of human serum albumin on membrane surfaces with atomic force microscopy. Here, they found that with increasing shear stress the hardness and the adsorption force of albumin increased, indicating that shear stress induced PVP elution may lead to increased protein adsorption on the membrane during dialysis treatment, which may then have negative implications on the hemocompatibility profile and performance characteristics of the membrane.

Low PVP elution should be an aim of dialyzer membranes both to maintain the benefits of increased hydrophilicity on the performance and hemocompatibility profile of dialyzer membranes during treatment, and to avoid the potentially deleterious effects of eluted PVP.

## 6. Discussion, Conclusions, and Future Directions

In summary, to improve well-being of the highly comorbid dialysis patients, good performance and hemocompatibility profile are two most important functions of a dialyzer. The membrane is the core component of the dialyzer and is mainly responsible for the performance characteristics and hemocompatibility profile. Through advances in material and production technologies, membranes are undergoing continuous development and refinements to become better replacements for the healthy human kidney. The pivotal characteristics of membranes are defined by their material, their membrane morphology and structure, including their pores and their blood-facing surface. Moreover, protein adsorption to the membrane strongly impacts both, the performance stability and the hemocompatibility profile of the dialyzer during dialysis treatment. Most synthetic membranes materials (e.g., PS, PES) are hydrophobic and are converted to hydrophilic by adding polyvinylpyrrolidone (PVP) to the membrane spinning mass. Increase in hydrophilicity reduces protein fouling and improves the hemocompatibility profile of the dialyzer, such as reduction in complement activation and decrease in platelet loss. To reduce the elution of PVP, α-tocopherol is added as an anti-oxidant and stabilizer to the spinning mass of novel membrane fibers.

Future studies need to investigate whether these improvements in membrane design will also result in long-term reduction in the chronic inflammation and cardiovascular burden of dialysis patients. Currently, a randomized controlled trial (eMPORA III, Comparison of Clinical Performance and Hemocompatibility of Dialyzers Applied During Post-dilution Online Hemodiafiltration, NCT04714281) is ongoing, which investigates performance and hemocompatibility of such a novel dialyzer over a longer period as compared to currently available clinical studies [37,42]. Moreover, given the potential positive impact of hydrophilic modification on the coagulation system, future clinical studies need to investigate whether such novel membranes are associated with improvements in the coagulation of dialysis patients, and may results in less need for anticoagulation. Finally, more experimental data are warranted to characterize structural features of modified membranes in more detail as well as the differences in amount, activation and type of proteins, adhering to different types of membranes.

Of note, while this review article is focusing on hydrophilic modifications of synthetic dialysis membranes and their positive impact on hemocompatibility and performance, also other membrane innovations may result in improvements of these two core properties of a dialysis membrane and improve the well-being of dialysis patients. Recent research with silicone nanopore membranes aims to develop an implantable hemofilter with selective solute permeability and good hemocompatibility. First experimental and animal studies showed promising results of such implantable hemofilter, also in terms of solute clearance and hemocompatibility [90,91,92]. Furthermore, while hydrophilic membranes may have the potential to reduce the need for anticoagulation during dialysis treatment, also other membrane modifications may lead to less need of anticoagulation. Early approaches used heparin-coated membrane surfaces [93,94,95,96], while recently a new hemodialyzer membrane modified with surface modifying macromolecules (SMMs) has been developed [97,98]. This membrane contains the fluorinated polyurethane SMM Endexo^TM^, which was designed to reduce protein and platelet adsorption [99]. Recent experimental data show that this novel membrane shows lower platelet adsorption and activation (15–60 min, *p* < 0.05) and higher clotting time (*p* < 0.05) as compared to a standard polysulfone dialyzer [97]. Moreover, data from a prospective clinical study comparing a standard polysulfone dialyzer (12 hemodialysis sessions) with the Endexo^TM^ dialyzer (38 hemodialysis sessions) demonstrated that while having a safe treatment profile, this dialyzer showed good performance (corrected mean β2-microglobulin removal rate was 47% higher during the Endexo^TM^ period) [98]. Finally, further research also focusses on the removal of larger toxins, such as protein-bound uremic toxins (PBUTs), which can be challenging with current dialysis membranes and modalities. The increase in pore size to increase the removal of such PBUTs face the problem of losing essential proteins, such as albumin [13,100]. Novel approaches try to remove such toxins by using PBUTs adsorbing multilayer membranes or other absorptive methods to remove these toxins from the patients’ blood [101,102,103].

In summary, hydrophilic modification of synthetic dialysis membranes is an effective way to improve performance and hemocompatibility, which are the two major features of a dialyzer membrane. Future research is needed to investigate how these improvements in performance and hemocompatibility will translate into long-term clinical benefits for patients with end-stage renal disease.

## Data Availability

Not applicable.
